# A recall-optimized LSTM–XGBoost ensemble model for river flood forecasting using multivariate time-series data—case study in Sri Lanka

**DOI:** 10.3389/frai.2026.1903150

**Published:** 2026-10-16

**Authors:** T. H. S. Sankalpa, G. H. C. Sandaruwan, A. M. Aslam Saja, N. T. M. Sajith, M. F. M. Ishqy

**Affiliations:** 1 Department of Electrical and Telecommunication Engineering, South Eastern University of Sri Lanka, Oluvil, Sri Lanka; 2 Business Continuity Management & Integrated Emergency Management Program, Faculty of Resilience, Rabdan Academy, Abu Dhabi, United Arab Emirates; 3 Department of Computer and Communication Systems Engineering, University Putra Malaysia, Serdang, Malaysia; 4 Department of Computer Science and Engineering, South Eastern University of Sri Lanka, Oluvil, Sri Lanka; 5 Department of Civil Engineering, South Eastern University of Sri Lanka, Oluvil, Sri Lanka

**Keywords:** cloud computing, early warning system, ensemble learning, flood forecasting, Internet of Things (IoT), water level monitoring, decision-level fusion, multivariate time series

## Abstract

Riverine (fluvial) flooding remains a significant climate-related disaster in many countries. Extreme rainfall events caused by climate change cause significant damage and disruption to communities living near riverbanks or neighboring land areas, particularly when risk-sensitive development is ignored. This necessitates an effective mechanism to predict river flooding events and provide timely early warnings to the most vulnerable populations in high-risk flood-prone areas. The dynamics of flood risks require a localized, automated, and scalable solution that can be centrally monitored. This study presents a river flood forecasting system integrating artificial intelligence (AI) and Internet of Things (IoT). This system was deployed in one of the flood-prone river basins in Sri Lanka to assess the operational feasibility of real-time hydrological monitoring and flood-risk classification. The proposed system employed two machine learning models, Long Short-Term Memory (LSTM) and Extreme Gradient Boosting (XGBoost), for flood-risk prediction and IoT sensors (rainfall and ultrasonic sensors) for real-time hydrological data collection. The machine learning models were developed using rainfall, discharge rate, and river water level data to predict flood-risk states. A rule-based ensemble strategy was implemented by combining the outputs of LSTM and XGBoost to prioritize positive-class detection and reduce missed positive-labeled observations. Model performance was evaluated primarily using recall for the positive flood cases, while the overall accuracy was reported as the complementary metric alongside positive-class recall, providing an overall assessment of classification performance. Among the evaluated input window configurations using the same chronological holdout, the rule-based ensemble model with a 1-day input window showed the most favorable observed balance of performance metrics, achieving 100% positive-class recall, with 98.73% accuracy, 50.00% positive-class precision, and 66.67% positive-class F1-score. The 100.00% recall was based on correctly classifying all four positive-labeled observations in the held-out test set (4/4; 95% exact binomial CI: 39.8%−100.0%) and these observations occurred consecutively from November 25 to 28, 2024, and represent one continuous positive-labeled flood episode. The proposed safety-oriented AI-IoT framework demonstrates the operational feasibility of integrating real-time sensing with AI-based flood forecasting; however, longer-term field deployment, including actual flood events, is required to evaluate flood-event prediction performance under real-world flood conditions.

## 1 Introduction

Flooding events account for a significant proportion of climate-related disasters, affecting millions of people annually and resulting in extensive loss of life, damage to infrastructure, and disruption of livelihoods (Stevance et al., 2025). Many developed and developing regions have become exposed to severe and frequent riverine floods due to rapid unplanned urbanization, land-use changes, and climate change-induced extreme rainfall (Paulose and Augustin, 2026). Disaster management authorities face major challenges in managing coastal flooding, flash floods, and river overflows, as these events have become increasingly difficult to predict. The impact of flood-related losses is exacerbated in low-lying and densely populated regions owing to limited preparedness and delayed warnings. A recent review on “Machine learning application in flood disaster” studies highlighted that advances in the latest technologies such as data collection using drones, satellites, and Internet of Things (IoT)-based sensors need to be explored in flood forecasting and monitoring (Uddameri and Hernandez, 2025).

Effective flood forecasting and early warning systems require real-time monitoring of river water levels accurately and continuously. Traditional flood monitoring approaches mainly rely on manual measurements and threshold-based sensor systems. These methods lack real-time data processing and analytical or predictive capabilities (Mostafiz et al., 2022). The latest developments in artificial intelligence (AI) models and real-time data acquisition techniques can potentially support the overcoming of these challenges (Agarwal et al., 2026). Machine learning (ML) models can be trained to handle complex hydrological data to capture nonlinear temporal patterns in rainfall-runoff and water-level dynamics (Guo et al., 2021). Accurate real-time data can be collected using sources such as Internet of Things (IoT) based sensors, satellite observations, and Unmanned Aerial Vehicles (UAV). Flood prediction systems can work with high accuracy and precision when the correct technologies are fused. The catastrophic impacts of river flooding under changing climatic conditions can be limited by proper early warning systems, informed decision-making, and enhanced emergency response plans that are sensitive to real-world conditions (Kim et al., 2023). While there are many cases where AI and ML models have been effectively used for flood forecasting and management, the integration of IoT further enhances a system's capability by enabling reliable, automated real-time data collection (Behnke and Austad, 2023).

Despite recent advances, several gaps remain in the integration of AI-based approaches for flood forecasting applications. First, relatively few studies have integrated IoT-based real-time sensing with AI models for operational flood forecasting (Ansari and Vidyarthi, 2025). Second, many AI-based flood prediction methods have been evaluated using only historical datasets, with limited validation under real-world deployments (Ansari and Vidyarthi, 2025; Perera et al., 2025). Third, while hybrid AI models have been investigated, only few models have focused on safety-oriented decision-level fusion strategies to minimize missed flood events (Frifra et al., 2024). Finally, AI-IoT-integrated flood forecasting frameworks remain limited within the Sri Lankan context, where the country is severely affected by flood disasters (Perera et al., 2025). This study attempts to address these gaps by developing and demonstrating the operational feasibility of a real-time IoT-enabled flood forecasting system that combines LSTM-XGBoost for a rule-based decision-level fusion strategy.

In this study, a real-time river water level monitoring and flood warning system is presented, where the system uses Long Short-Term Memory (LSTM) and Extreme Gradient Boosting (XGBoost) models combined through a rule-based decision-level fusion approach for flood-risk classification while minimizing false negatives. The prediction system was fed with real-time river water level, rainfall level data via IoT sensors, and catchment water release data through public data sources. Moreover, the proposed system was designed to be cost-effective, energy-efficient, and autonomous, utilizing solar power and wireless communication for sustainable remote operation. A flood in this study refers to “river flood” (also referred to as “riverine” or “fluvial” flooding) is defined as “the water overflow from stream channel onto normally dry land in the flood plain” (Adil et al., 2025). Existing flood monitoring methods in Sri Lanka often depend on manual data collection or static threshold systems that do not incorporate predictive analytics. Therefore, flood forecasting methods supported by data-driven approaches need to be tested extensively to improve evidence-based decision making in smaller nations such as Sri Lanka with abundant rivers.

The system developed in this study was field-tested in the river flood-prone downstream regions of the Gal Oya River in the Eastern part of Sri Lanka. The study had four key objectives:

- Development of LSTM and XGBoost models for flood risk classification and forecasting
- Integration of the models into a rule-based ensemble framework to test their reliability
- Development of a solar-powered IoT-enabled water level monitoring kit for real-time data collection
- Development of a cloud-based dashboard for data visualization and alert dissemination

The findings of this study demonstrate the operational feasibility of an intelligent flood forecasting system evaluated under local conditions in Sri Lanka. The proposed framework provides the basis for future investigation, adaptability, and scalability to other river basins and flood-prone regions in Sri Lanka and other regions.

## 2 Literature review

This section reviews recent works on AI-based early flood warning systems. The approach taken here initially reviews the contribution of ML models and AI approaches in flood predictions. The focus is then given on the key data sources and variables used to characterize flooding events. Then, a review is conducted to identify suitable models to train for flood forecasting and flood risk level classification that utilizes identified key parameters. The review then discusses an ensemble approach for increasing the accuracy of predictions. Finally, the section discusses the existing contributions of IoT in flood forecasting systems. This approach to the literature review provided a systematic understanding of the existing approaches while identifying clear research gaps.

### 2.1 Machine learning and AI approaches

The main function of AI-based flood predicting models is to generate early warnings (Miiro et al., 2025) based on available historical and real-time sensor data (Nihal et al., 2024). Such systems are expected to process data and disseminate early warnings to communities to increase resilience against flood events.

The analysis of hydrological data remains the core of flood prediction systems because it is required to capture the nonlinear, dynamic, and spatiotemporal relationships inherent in hydrological processes (Xiang, 2025). Data-driven machine learning approaches, such as random forest and decision trees, have been widely applied to model rainfall and river flow data, helping to predict overflow trends (Perera et al., 2025). These methods are simple and require fewer computational resources than deep learning, while providing reliable performance when sufficient data are available. However, there is a trend toward adopting advanced data-driven methods, particularly long short-term memory (LSTM) and extreme gradient boosting (XGBoost), to improve the accuracy, reliability, and real-time adaptability of flood forecasting systems (Fang et al., 2021; Waleed and Sajjad, 2025). The reviewed literature collectively illustrates that these models have demonstrated outstanding performance in handling complex datasets and generating accurate forecasts, thereby contributing significantly to flood risk classification and forecasting decision-making.

#### 2.1.1 Key data sources and variables for accurate flood forecasting

The accuracy of flood forecasting can be improved by understanding local environmental, geographical, and climate conditions in addition to hydrological data. For example, in Sri Lanka, the rainfall intensity during the southwest monsoon season remains a primary factor for flooding events (Prabuddhi and Seneviratne, 2020). Real-time and historical data, including rainfall catchment data, are essential for understanding basin-wide water inflow (Wijayaraja et al., 2024). Critical factors to identify river flooding risks include river water levels (Perera, 2024), reservoir water levels (Krishly et al., 2020), and water flow speeds (water discharge rate) (Ilukkumbure et al., 2021).

Furthermore, topographical factors, such as terrain and urban development, influence flood vulnerability. Low-lying urban areas with poor drainage systems and reduced wetlands are particularly prone to flooding (Herath et al., 2023). Environmental conditions, such as high humidity and low evaporation, prevent floodwaters from receding quickly, worsening the flood situation (Wickramasooriya and Ravihari, 2025). The effectiveness of combining local hydrological data with external meteorological inputs has been highlighted as a key factor in building highly accurate AI-driven flood forecasting systems (Wang and Abdelrahman, 2023).

Some research studies on flood modeling have utilized diverse input parameters, including precipitation, water level, river discharge, temperature, evapotranspiration, wind speed, wind gust, slope, soil type, and land-use or flood map data (Avila-Aceves et al., 2023). It is understood that when models are trained with these parameters, they are better able to capture both temporal and spatial variations. While the importance of rainfall and discharge are emphasized as key drivers for reservoir inflow and flood occurrences (Perera et al., 2025), the value of combining meteorological and hydrological parameters for improved flood hazard forecasting and classification is also highlighted (Kim and Kim, 2021).

### 2.2 AI-based model for flood forecasting

The LSTM model is a special form of recurrent neural network (RNN) capable of capturing long-term temporal dependencies in time series and addressing the vanishing gradient problem (Hochreiter and Schmidhuber, 1997). Since events leading to flooding can be presented as time series data, LSTM networks are widely used for flood forecasting. One study in Sri Lanka applied LSTM to predict water levels based on rainfall and reservoir inflow data, where the model successfully captured long-term dependencies and accurately predicted water levels even during extreme weather events (Prabuddhi and Seneviratne, 2020). In such a case, LSTM achieved a coefficient of determination (R^2^) of 0.8710 and a root mean square error (RMSE) of 0.0854, significantly outperforming the multi-layer perceptron (MLP) model (*R*^2^ = 0.7206, RMSE = 0.2169). Another study supported evacuation planning by forecasting flood peaks several hours ahead using LSTM (Krishly et al., 2020). Both studies reported that LSTM outperforms simple regression models because it understands the sequence and delay between rainfall and resulting floods. These studies have demonstrated that LSTM outperforms traditional models in both short - and long-term forecasting horizons.

#### 2.2.1 AI-based model for flood risk classification

A study on flood risk classification models categorized water levels into risk groups, such as residual, low, moderate, significant, and extreme (Maranzoni et al., 2023). Common models, such as random forest, decision trees, support vector machines (SVM), and gradient boosting, can be used for risk classifications (Kim et al., 2023). Decision trees are often preferred for situations with limited data because they are simple, easy to train, and provide reasonable accuracy without large datasets. These classifications trigger alerts and assist authorities in making quick decisions.

The XGBoost algorithm is an ensemble learning method based on gradient-boosted decision trees (Chen and Guestrin, 2016). A review identified XGBoost as a leading ML method for flood risk classification and rainfall–runoff modeling (Uddameri and Hernandez, 2025).

In a flash flood risk assessment study (Ma et al., 2021), XGBoost showed the highest accuracy among other indices, indicating its robustness in classifying diverse hydrological conditions. XGBoost has a built-in feature that can be used to identify the input parameters that most strongly influence the results of the model. Hence, the application of this specific feature in XGBoost may provide useful information for-decision-making in hydrological applications.

#### 2.2.2 Combination of forecasting and classification models

Some studies have suggested that an ensemble-model approach is preferable for flood prediction as overfitting can affect safety, cost, and trust (Berkhahn et al., 2019). The ensemble approach utilizes the results from one model to predict water levels and another to classify the flood risk based on the predicted output. A study used an LSTM model for water-level forecasting and a Random Forest for flood risk classification (Kim et al., 2025).

Among the reviewed models, LSTM and XGBoost consistently outperformed other techniques, such as multiple linear regression (MLR), support vector regression (SVR), decision tree (DT), random forest (RF), and MLP (Kuhaneswaran et al., 2025; Uddameri and Hernandez, 2025). Each of these algorithms offers distinct advantages; however, the deep-learning architecture of LSTM and ensemble tree-based architecture of XGBoost make them particularly well-suited for hydrological time-series data, which are often noisy, nonlinear, and influenced by multiple interacting variables.

In addition to model selection, several studies have emphasized the importance of data preprocessing and feature engineering to enhance model performance. Techniques such as normalization, log transformation, and stationarity testing using the Augmented Dickey-Fuller (ADF) test are commonly applied to ensure stable data distributions (Tawakuli et al., 2025). In flood prediction, time features such as sine and cosine transformations have been introduced to capture seasonal variations in rainfall and discharge patterns, which have been found to substantially improve the accuracy of models such as LSTM and XGBoost by reducing noise and enabling the algorithms to better learn underlying temporal patterns (Kuhaneswaran et al., 2025; Uddameri and Hernandez, 2025).

A review on the combined applications of geographic information systems (GIS) with AI models explored how technology integration increases environmental intelligence and proactive risk governance, including flood predictions (Sarker and Jahan, 2025). A study in the Sri Lanka Nilwala River Basin demonstrated how AI can support both forecasting and emergency planning by using GIS and a multi-layer perceptron (MLP) neural network to process flood-related factors, such as slope, flow direction, and soil type, to classify flood-prone and safe zones (Perera et al., 2025). The study used Dijkstra's algorithm to identify optimal evacuation routes. While MLP models can provide satisfactory classification accuracy. Other studies showed that LSTM-based models achieve lower error metrics such as RMSE, Mean Absolute Error (MAE), and Nash-Sutcliffe Efficiency (NSE), indicating their greater suitability for hydrological forecasting tasks that involve temporal variability (Krishly et al., 2020; Prabuddhi and Seneviratne, 2020).

Our review identified LSTM and XGBoost to be among the most widely adopted and high-performing models for flood and water level forecasting. While LSTM is effective for learning temporal dependencies from sequential hydrological data, such as rainfall, discharge, and reservoir inflow represented as a time series, XGBoost provides high interpretability, strong generalization capability, robust classification accuracy, and feature-based decision making for analyzing complex feature interactions. The findings from the review support the selection of LSTM and XGBoost as candidate models for the study presented in this paper.

Furthermore, a literature review revealed the growth in the use of hybrid and ensemble modeling, where deep learning and boosting algorithms are integrated to exploit their respective strengths. Some hybrid models, such as LSTM–autoregressive integrated moving average (ARIMA) (Xu et al., 2022) and LSTM–XGBoost (Frifra et al., 2024), have shown increased performance in terms of accuracy and reliability over single-model approaches. Such hybrid systems can be very suitable for flood forecasting frameworks as short-term memory and long-term consistency are essential. The ensemble of LSTM and XGBoost models can effectively balance each other.

This study aimed to combine these models to develop a rule-based ensemble strategy framework capable of operating with real-time data. This approach draws on the strengths identified in previous research and provides a basis for developing a flood forecasting system adapted to the hydrological characteristics of the study location. Although recent architecture such as Transformer-based models, gated recurrent unit (GRU), and convolution neural network (CNN)-LSTM have shown promising results in time-series forecasting, our study did not include them because of availability of a relatively small and highly imbalanced dataset. Transformer and hybrid deep learning architectures generally involve greater model complexity and require larger datasets and careful regularization. However, LSTM and XGBoost have been widely applied in hydrological forecasting, and their characteristics were considered suitable for the scope of this study.

### 2.3 IoT integration and real-time data collection

IoT systems enable data acquisition from local sensors, communication with cloud platforms, and interfacing with legacy industrial systems using Modbus TCP/IP or serial communication, facilitating scalable, flexible, and secure IoT deployments in industrial environments (Boyes et al., 2018). The integration of IoT has greatly streamlined smart monitoring solutions that collect and transmit real-time data from various sensors. A critical review of 166 research articles on the use of IoT in water resources applications revealed that more research is needed on advanced IoT and AI/ML integration to accurately assess flood risk and devise rapid disaster response preparedness strategies, as only 13% of the articles either tested IoT or AI/ML for flood monitoring and forecasting (Ansari and Vidyarthi, 2025). Table 1 summarizes a detailed comparison of the novelty and methodology of the proposed framework against two similar studies (Xu et al., 2022; Frifra et al., 2024).

**Table 1:** Comparison of methodology of proposed framework with similar hybrid approaches.

Feature	Xu et al. (2022)	Frifra et al. (2024)	Proposed framework
Core system	Sequential Pattern Separation- mathematically separate linear patterns	Dual-task parallelism by running models in unison to predict different aspects of the same event	Combines a physical hardware layer with cloud-based AI prediction layer
LSTM	Predicts residual/nonlinear time series	Predicts multiple storm characteristics	Predicts future river water level
XGBoost	Not used for event classification	Classifies storm occurrences from predicted storm variables	Independently classifies flood occurrences
Relationship of the models (Ensemble logic)	LSTM model is trained specifically to predict the errors of the ARIMA model	The models act independently on the same dataset to provide a holistic view of the storm	Two independently trained models fused at decision level: If either model predicts a positive risk the system triggers a warning to prioritize positive-class detection (safety-first)
Fusion	Residual modeling	Regression + classification	Decision level fusion (OR logic safety first rule)
Output	Drought index	Storm occurrences	Flood warning
Data sources	Large-scale historical records	Multivariate marine records (long-term buoy measurements with a storm database)	Hybrid real-time sourcing (integrated custom deployed IoT node with public hydrological APIs)
Evaluation / Demonstration	Using historical data	Using unseen historical data	Predictive evaluation using historical labeled data and operational feasibility demonstrated using real-time field deployment

## 3 Case study details: rationale for selecting Sri Lanka's Gal Oya river flooding

Flooding in Sri Lanka has caused average annual losses of US$240 million and affected more than 10 million people in the past three decades (Group, 2021). Floods are the most frequent and highly destructive disaster events among the 25,000 extreme weather-related events reported between 1975 and 2022 (Group, 2025). In the past, flood disasters in Sri Lanka resulted in loss of life, extensive damage to the property, and severe disruption to the country's socio-economic performance. The Climate Risk Index for 2016 by Germanwatch ranked Sri Lanka fourth among the top 10 most affected countries globally (Eckstein et al., 2018). In recent years, increasing intensity of rainfall events driven by climate change has worsened the flood situation in Sri Lanka, which has become an annual event, particularly in river basins such as Deduru Oya and Gal Oya.

In the Deduru Oya and Nilwala river basins of Sri Lanka, flood forecasting systems have been deployed to monitor water levels, and the collected data are manually processed to provide early warnings to facilitate evacuation and disaster-response efforts (Krishly et al., 2020; Perera et al., 2025; Prabuddhi and Seneviratne, 2020). Table 2 presents the details of the input parameters, methodology, and outputs obtained from the three AI-based studies in the two river basins in Sri Lanka.

**Table 2:** Review of three selected AI-based approaches related to water level forecasting and flood risk classification in Sri Lanka.

References	Inputs	Methodology	Outputs/Results	Limitations
Perera et al. (2025)	Rainfall - Water level Water discharge Slope and water flow direction Soil Type Flood map data	Processed six flood-related parameters using GIS. Used an MLP for flood-prone area classification. Applied Dijkstra's algorithm for evacuation route planning. Trained LSTM model to predict reservoir inflow using rainfall and inflow history The evaluation was performed using MSE, RMSE, MAE, and NSEf.	Forecasted reservoir inflow for future time steps Generated flood hazard maps. LSTM outperformed ANN and MLP, with the lowest error values.	The accuracy of predictions using Meteo API data depends on the quality and availability of the data. Models may require refinement as the weather patterns change.
Krishly et al. (2020)	Precipitation Wind speed Wind gust Evapotranspiration River discharge Temperature	Data collected from six rainfall and one hydrological stations The ADF test was applied for the stationarity check. Log transformation was performed to correct skewness. Sine/cosine time features were added for seasonality. Trained SVR, MLR, Random Forest, and XGBoost models for 7-day discharge forecasting	Accurate rainfall and discharge prediction models were built. XGBoost and Random Forest achieved the highest accuracy. High prediction performance at Deniyaya and Kekanadura stations A 7-day river discharge forecasting system was developed.	Limited dataset size The model comparison may not be fully reliable due to possible imbalance in parameter tuning Lacks validation under operational or real-time conditions
Prabuddhi and Seneviratne (2020)	Rainfall Temperature Evaporation Water level	LSTM and MLP models were developed for flood prediction. Compared stochastic gradient descent (SGD) and Adam optimizers (Adam performed better). An 80/20 data split with normalization was used. Combined LSTM + ARIMA for hybrid long-term forecasting Classified water levels into four flood categories: no flood and major flood.	LSTM: *R^2^* = 0.8710, RMSE = 0.0854. MLP: *R^2^* = 0.7206, RMSE = 0.2169. Hybrid LSTM–ARIMA: *R^2^* = 0.8901, RMSE = 0.0247. Predicted flood levels were predicted up to 300 days ahead. LSTM significantly outperformed the MLP.	Lack of data & missing values No real-time system Data-quality limitations Limited data availability

The review of these three models indicated that LSTM outperformed MLP and artificial neural network (ANN) in two studies, while XGBoost and RF achieved the highest accuracy in the respective studies. However, all three studies lacked to demonstrate operational feasibility or validation with real-time conditions, and in some cases, had limited dataset size or missing data values. Similar studies using AI-based models for water level monitoring and flood risk classification were conducted in the Philippines (Kim et al., 2023) and Korea (Kim and Kim, 2021). These two studies used LSTM, deep neural network (DNN), SVM, and XGBoost to train the models. However, they used smaller datasets to forecast water levels and classify flood risks. Furthermore, they did not use hybrid or ensemble modeling.

In practice, traditional flood monitoring systems in Sri Lanka rely heavily on manual observations and basic threshold-based alert mechanisms, which often fail to provide timely and accurate warnings. These systems also lack integration with advanced data analytics and real-time monitoring capabilities of emerging digital technologies, limiting their effectiveness in supporting disaster preparedness and early warnings. Therefore, it is important to explore the potential of an intelligent, real-time, and low-cost system that can accurately predict river flood events using the available AI and IoT technologies.

Figure 1A shows the major rivers in Sri Lanka, including Gal Oya, with the inset map of the Gal Oya River Basin (location of this study). The entire Gal Oya basin has not been frequently flooded in the past because of the high capacity of the Senanayake Samudraya Reservoir. For example, as shown in Figure 1B, the 2011 inundation map of the Gal Oya basin shows that only certain areas were affected by the flood. However, it was revealed that there has been an increasing trend of flood events during the past four decades (1976–2018), and this trend is expected to continue in the future (2040–2059) as evidenced from the models developed for the Gal Oya River basin (Ketipearachchi, 2021; Zuhail, 2019). Therefore, there is an urgent need to enhance the effectiveness of flood forecasting to make well-informed evidence-based decisions.

**Figure 1:**
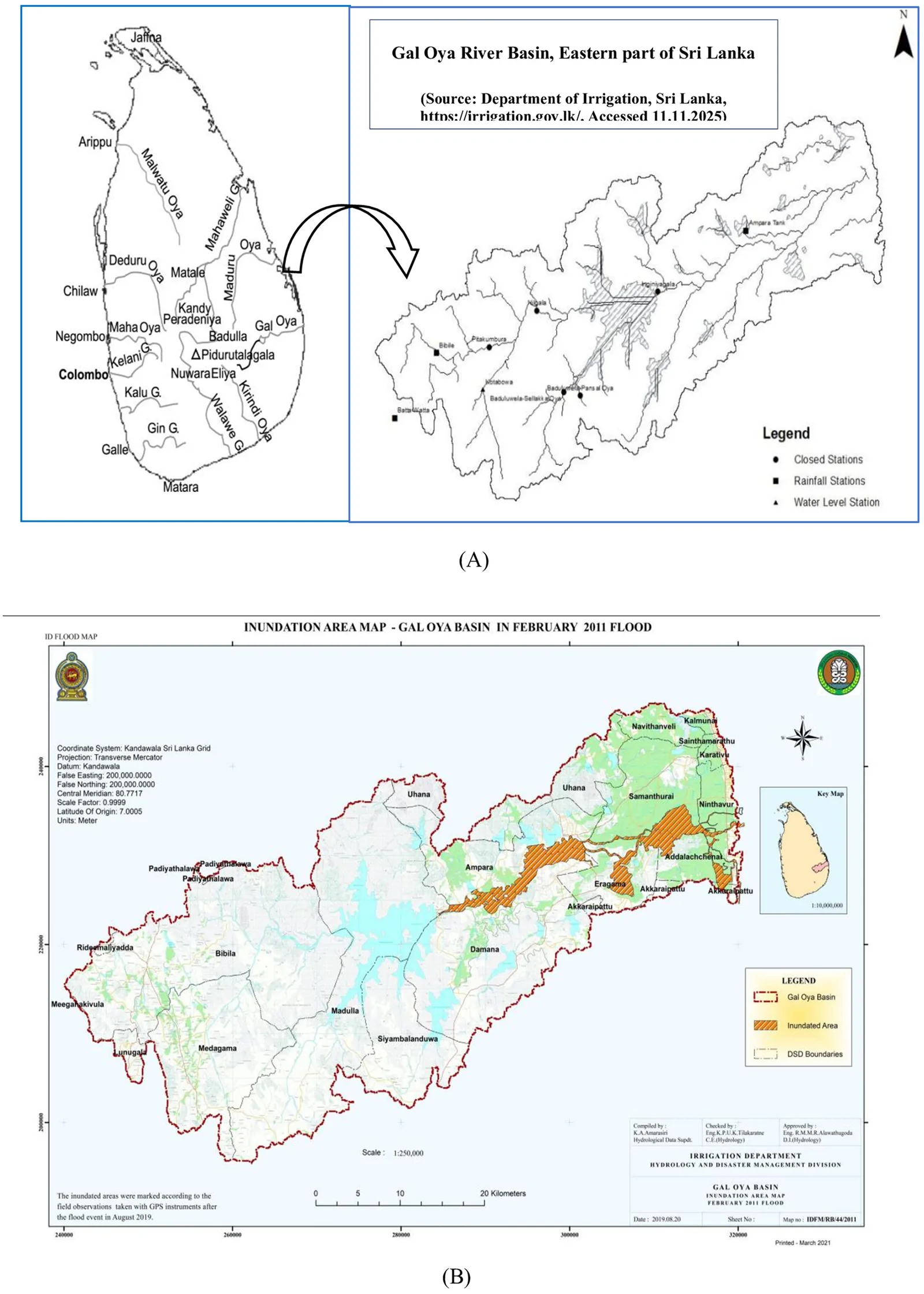
**(A)** Gal Oya River basin and its location in Sri Lanka map. **(B)** Inundation area map – Gal Oya basin due to 2011 food.

## 4 Study methodology

This section details the methodology adopted to design and develop an AI-IoT-based flood level classification and forecasting using river water-level monitoring. The proposed system architecture comprises an IoT-based hydrological data acquisition layer, an AI-based prediction layer comprising LSTM and XGBoost models, and a data visualization layer. The methodology encompasses IoT system development, data acquisition and preprocessing, feature selection, model development, training, and validation processes.

### 4.1 System architecture overview

The proposed system architecture is designed to enable continuous monitoring of hydrological conditions in the selected locations to make timely and accurate flood forecasts. As shown in Figure 2, the system integrates sensing, communication, data processing, and decision-support components to transmit rainfall and water-level data in real-time and generate actionable observations. The architecture adopts a modular and scalable architecture to ensure seamless data collection from field-deployed sensors to a central analysis layer for real-time monitoring as well as historical data analysis for proper flood management.

**Figure 2:**
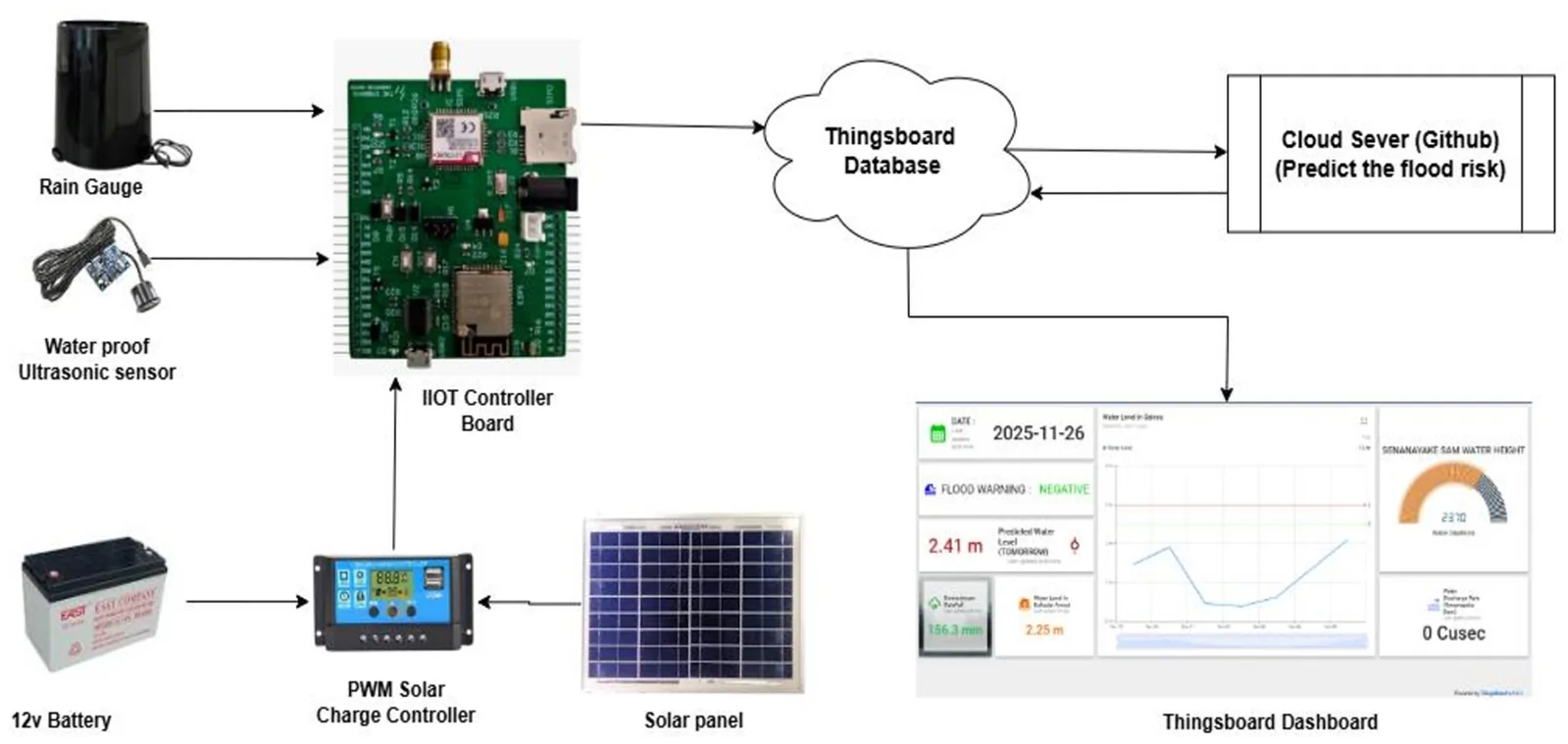
Overview of the system architecture.

#### 4.1.1 Data acquisition and preprocessing

The development of the proposed AI-based water level monitoring and flood forecasting system included both real-time and historical hydrological data collected from the Gal Oya river basin in Sri Lanka. The dataset includes key environmental parameters, such as catchment rainfall data, downstream rainfall data, river water level, dam water level, and dam water discharge rate, which are critical indicators of flood risk. While the real-time data were collected using IoT-field devices to demonstrate the feasibility of continuous environmental monitoring under real-world conditions, the historical data for model training were obtained from the Department of Irrigation and the Department of Meteorology of Sri Lanka.

Real-time sensor data are transmitted to the data processing layer by the IoT controller board, which utilizes the NB-IoT or Wi-Fi protocol for connectivity, depending on network availability. The remotely deployed sensor and controller are powered by a 12 V solar panel connected to a solar controller to ensure reliable connectivity, even in off-grid environments. The transmitted numerical data are directly presented on the browser-based dashboard, while the AI layer decision based on the real-time data is also displayed.

The field deployment was intended to demonstrate the operational feasibility of the proposed IoT monitoring architecture, including the sensor operation, real-time data acquisition, data transmission, and visualization under field conditions. While the field data was used during the operational demonstration, the predictive performance of the proposed machine learning models was evaluated using official historical datasets.

Raw historical data spanning from 2020/03 to 2025/04 (approximately 5 years, comprising 1,873 daily observation entries) were preprocessed to create the training dataset. After model training, the same preprocessing pipeline was applied to all validation, testing, and real-time input data before inference. This ensured consistency in preprocessing between model training and the real-time inputs used for the operational demonstration. The data preprocessing steps included:

- Data cleaning: Numeric columns were converted to common formats, while removing missing values. Sensor data were also subject to cleaning to eliminate duplicate readings due to any communication glitches.
- Threshold selection: The historical hydrological data used in this study were obtained from the Department of Irrigation, Sri Lanka, while independent evidence of historical flood conditions was retrieved from public official flood-warning records for the Gal Oya Basin.^1^ The historical flood conditions were first identified independently using the available official flood-warning/event records before the threshold value was determined. The observed river water-level records corresponding to these documented flood conditions were then examined to identify a practical local water-level threshold that consistently represented the observed flood conditions at the study location.
  The analysis concluded that 3.0 m is an appropriate local threshold for the purpose of this study as recorded river water-level during the flood events were minimum 3.0 m. The threshold represents an empirically identified local reference for defining the binary target variable for this study (Equation 1), which is used for labeling.
$$\begin{array}{l} Flood=\;\{\begin{array}{c} 1,Water\;Level\geq 3m\\ 0,\;Water\;Level\;<3m \end{array} \end{array}$$ (1)
  Where: *Flood* is the flood risk level.
  The dataset available for this study did not include a threshold value based on a formal national vertical datum or surveyed gauge datum. Hence, the threshold value was reviewed by a civil engineer in the research group with knowledge of the local hydrological and river conditions. For this study, 3.0 m was taken as a practically suitable level for local flood labeling based on the available evidence as explained above.
- Labeling: Categorical flood risk levels were converted into numeric labels for the XGBoost classification, and assignment of categorical flood risk labels (POSITIVE or NEGATIVE) based on the identified water level threshold to support the rule-based ensemble decision logic. In this study, a positive sample or flood-labeled sample refers to an individual daily observation for which the flood-risk label is POSITIVE. Multiple consecutive positive samples correspond to the same continuous flood episode. The available historical dataset consists of two periods of continuous positive-labeled observations: January 11-15, 2024, and November 25-28, 2024. Accordingly, the nine positive-labeled observations represent two continuous positive labeled flood episodes in the historical dataset.
- Feature Engineering: This included the selection of essential input parameters, including catchment rainfall, downstream rainfall, reservoir water level, reservoir discharge rate, and river water level, which directly influence flood occurrence.
- Normalization: Scaling of all feature values and target water levels to a range between 0 and 1 using min-max scaling was adopted to enhance model training efficiency and convergence. The normalized value was computed as shown in Equation 2.
$$\begin{array}{l} x_{norm}=\;\frac{x-x_{\min}}{x_{\max}-x_{\min}} \end{array}$$ (2)
  Where: *x* is the original value, *x*_min_ *and x*_max_ are the minimum and maximum values of the corresponding feature in the training dataset.

The finalized dataset was split as 80/20, maintaining a strict chronological holdout strategy, for training and testing sets. No random shuffling was performed so that the temporal ordering of the time-series was preserved, and future observations in the chronological holdout were not used for model training. The XGBoost training dataset was subjected to the Synthetic Minority Oversampling Technique (SMOTE) to balance flood risk classes. Furthermore, the model training was conducted only after statistical and correlation analyses to identify relationships between rainfall and water level variations. This systematic data acquisition and preprocessing approach provided a consistent basis for fitting the LSTM and XGBoost models using the chronological training portion and subsequently evaluating their observed performance on the chronological holdout. These modeling choices were only based on the characteristics of the problem, domain knowledge, literature, and available training data. The chronological holdout was not inspected or considered when developing these models and selecting their training parameters.

### 4.2 AI model development

The AI model development process involved designing and training three distinct modeling approaches using the chronological portion of the historical hydrological data, followed by the evaluation of the developed modeling framework under varying temporal input-window configurations. The input features for all models included the catchment and downstream rainfall, river and dam water levels, and the dam water discharge rate. The models were trained on 80% of the data and evaluated using the remaining 20%.

#### 4.2.1 Temporal alignment of predictor variables and prediction targets

A one-step-ahead forecasting formulation was employed to temporally align the predictor variables and prediction targets to ensure that the prediction models used only information available at the forecast origin. The forecast origin, denoted by *t*, represents the completed daily observation period at day *t*. The forecast for the next day, *t* + 1, is generated using the information available up to the forecast origin. For an input window of *w* days, the predictors comprise the chronologically ordered observations from day *t – w* + 1 through day *t*, which are used to predict the target at day *t* + 1. Hence, the input window size represents the amount of historical information provided to the model.

Further, the forecast lead time remains 1 day for all input window configurations. The catchment rainfall used in the operational system was implemented to retrieve the total precipitation for the preceding day using the weather API. Thus, the catchment-rainfall value used in the operational forecast represents a completed daily rainfall observation rather than an incomplete rainfall accumulation from the forecast day. The historical rainfall records used for model development were similarly represented as daily rainfall observations in millimeters. The downstream rainfall and river water level were obtained through the field sensor feed at the time of inference. The predictor variables consisted of chronologically ordered observations at time *t*, including catchment rainfall, downstream rainfall, reservoir water level, reservoir discharge rate, and Kaliodai river water level. The prediction target was obtained from the subsequent observation at time *t* + 1.

For the LSTM model, the predictor observation at time *t* was used to predict the Kaliodai River water level at the subsequent time step, *t* + 1. The temporal relationship is defined as shown in Equation 3:


$$\begin{array}{l} X_{t}\to {Ĥ}_{t+1} \end{array}$$ (3)

Where: *X*_*t*_ represents the predictor variables at time *t*, and Ĥ_*t*+1_ represents the predicted Kaliodai River water level at the subsequent time step *t* + 1.

The observed river water level at *H*_*t*+1_ was not included in the predictor vector for that instance and was used as the ground truth target during model training and evaluation.

For the XGBoost classifier, the predictor variables available at time *t* were paired with the flood-risk target at the subsequent time step *t* + 1. This relationship is defined as shown in Equation 4:


$$\begin{array}{l} X_{t}\to {Ŷ}_{t+1} \end{array}$$ (4)

Where: Ŷ_*t*+1_ represents the predicted flood-risk class at the subsequent time step *t* + *1*.

The flood-risk target (Equation 5) was determined from the observed Kaliodai river water level at *t* + 1 using the 3.0 m local flood-labeling threshold value described in Section 4.1.1.


$$\begin{array}{l} Y_{t+1}=\;\{\begin{array}{c} 1,\;H_{t+1}\geq 3\;m\\ \;0,\;H_{t+1}<3\;m \end{array} \end{array}$$ (5)

Although the current Kaliodai River water level *H*_*t*_ may be included among the predictor variables at the prediction time, future water level *H*_*t*+1_, which determines the future flood-risk label, is not included in the predictors. Hence, the water-level observation associated with the target flood state is not provided to the models when the prediction is generated. The same temporal ordering was maintained throughout the model construction. The training and testing data were separated chronologically to ensure that future test observations were not used as predictor observations for training samples.

For the operational requirement, the next-day forecast is generated daily as scheduled in the prediction workflow. The workflow was scheduled at 18:15 UTC (23:45 Sri Lanka Standard Time) and retrieved the predictor values immediately before the inference. Catchment rainfall represents the completed daily precipitation total for the preceding calendar day. Downstream rainfall measured is the accumulated daily total rainfall, which is reset at midnight. Kaliodai River water level is an instantaneous measurement from the IoT node at the forecast run. Reservoir water level and reservoir discharge are retrieved as the currently available values from the Irrigation Department of Sri Lanka data sources at the forecast run. The five predictor values are then supplied to the LSTM and XGBoost models to generate the forecast for the subsequent day. The scheduled execution time represents the forecast data retrieval and forecast time rather than the measurement time of the upstream reservoir variables. Table 3 specifies the temporal representation, source, and availability of each predictor.

**Table 3:** Predictor-specific details of temporal representation, source, and availability of each predictor.

Predictor	Temporal representation	Availability / Collection in operational system
Catchment rainfall	Calendar-day accumulated rainfall	Retrieved from Open-Meteo historical API at the scheduled forecast workflow run
Downstream rainfall	Calendar-day accumulated rainfall (reset at midnight)	Retrieved from the IoT platform at the scheduled forecast workflow run
Kaliodai River water level	IoT water-level telemetry value (measured instantaneously)	Retrieved from the IoT platform at the scheduled forecast workflow run
Reservoir water level	Currently available upstream source (API) value	Retrieved from the Irrigation Department API during the scheduled forecast workflow run.
Reservoir discharge	Currently available upstream source (API) value	Retrieved from the Irrigation Department API during the scheduled forecast workflow run.

#### 4.2.2 Method 1: LSTM for water level forecasting

This approach employs an LSTM network to forecast the continuous numerical river water level. A flow chart of the process is shown in Figure 3.

- Forecasting and classification: The LSTM model, which is effective for time-series data, was configured as a one-step-ahead forecasting model to use the predictor observations available at time *t* to estimate future water levels at the subsequent time step *t* + 1. This numerical output was then compared with the predefined threshold to indirectly classify the flood risk as either “negative” (safe) or “positive” (risk).
- LSTM model parameters: The LSTM model consisted of two stacked LSTM layers containing 64 and 32 hidden units, respectively. A fully connected dense layer consisting of 16 neurons and ReLU activation was used before the final output layer. The model was trained using the Adam optimizer with a learning rate of 0.001 and mean squared error (MSE) as the loss function. Furthermore, the training used a batch size of 16 for 50 epochs using a 10% validation split. To examine the effect of historical input length, input window configurations of 1, 7, 14, and 21 days were evaluated while keeping the model architecture and hyperparameters unchanged. For each input window, the corresponding sequence of daily predictor observations ending at day *t* was used to predict the river water level at day *t* + 1.
- LSTM evaluation metrics: LSTM regression model performance was evaluated using RMSE, MAE, R^2^, and NSE using Equations 6–9. The values of these parameters are provided in the Section 5.

$$\begin{array}{l} RMSE=\;\sqrt{\frac{1}{N}\overset{N}{\underset{i=1}{\sum }}{\left(y_{i}-{ŷ}_{i}\right)}^{2}\;} \end{array}$$ (6)

$$\begin{array}{l} MAE=\;\frac{1}{N}\overset{N}{\underset{i=1}{\sum }}|y_{i}-{ŷ}_{i}| \end{array}$$ (7)

$$\begin{array}{l} R^{2}=1-\frac{\sum_{i=1}^{N}{\left(y_{i}-\hat{y_{i}}\right)}^{2}}{\sum_{i=1}^{N}{\left(y_{i}-\bar{y}\right)}^{2}} \end{array}$$ (8)

$$\begin{array}{l} NSE=1-\frac{\sum_{i=1}^{N}{\left(y_{i}-\hat{y_{i}}\right)}^{2}}{\sum_{i=1}^{N}{\left(y_{i}-\bar{y}\right)}^{2}} \end{array}$$ (9)

Where: *y*_*i*_ denotes the observed water level, ŷ_*i*_ denotes the predicted water level, $\hat{y_{i}}$ denotes the predicted water level, $\bar{y}$ denotes the mean of the observed water level, and *N* denotes the total number of observations.

**Figure 3:**
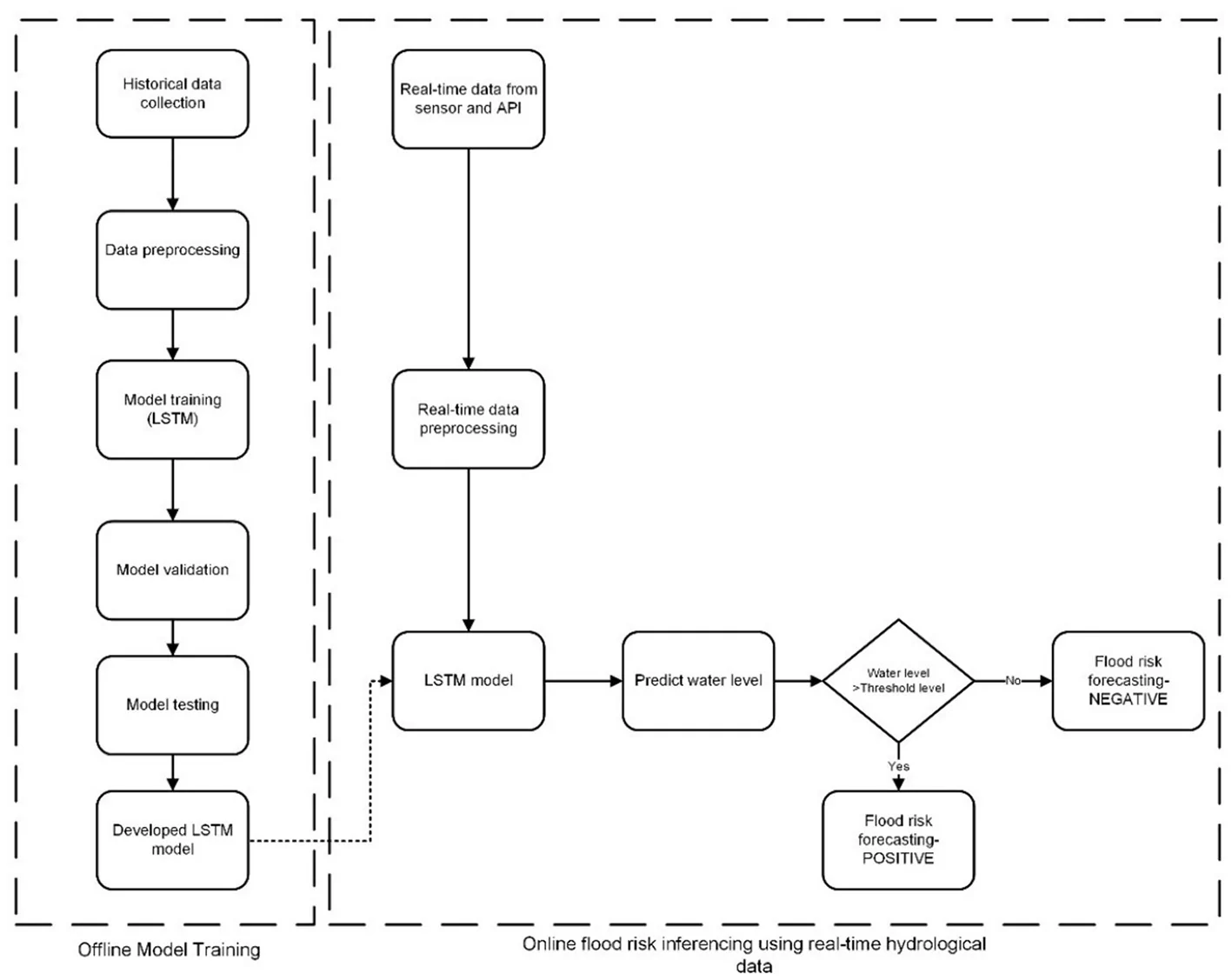
Single-model (LSTM) water level forecasting.

#### 4.2.3 Method 2: XGBoost for direct risk classification

This method used the XGBoost classifier to predict the flood risk category directly. A flow chart of the process is shown in Figure 4A.

- Direct classification: Instead of forecasting the water level first, the XGBoost model directly classifies the flood-risk state at the subsequent time step *t* + 1 using the predictor variables available at the prediction time *t*. Although the Kaliodai River water level at time *t* was included as one of the predictor variables, the river water level at *t* + *1*, which was used to determine the flood risk state, was not included in the predictors.
- Imbalance handling: Because the historical data was severely imbalanced (few “positive” flood cases), the SMOTE (Synthetic Minority Over-sampling Technique) was applied to the training set to generate synthetic minority class samples.

**Figure 4:**
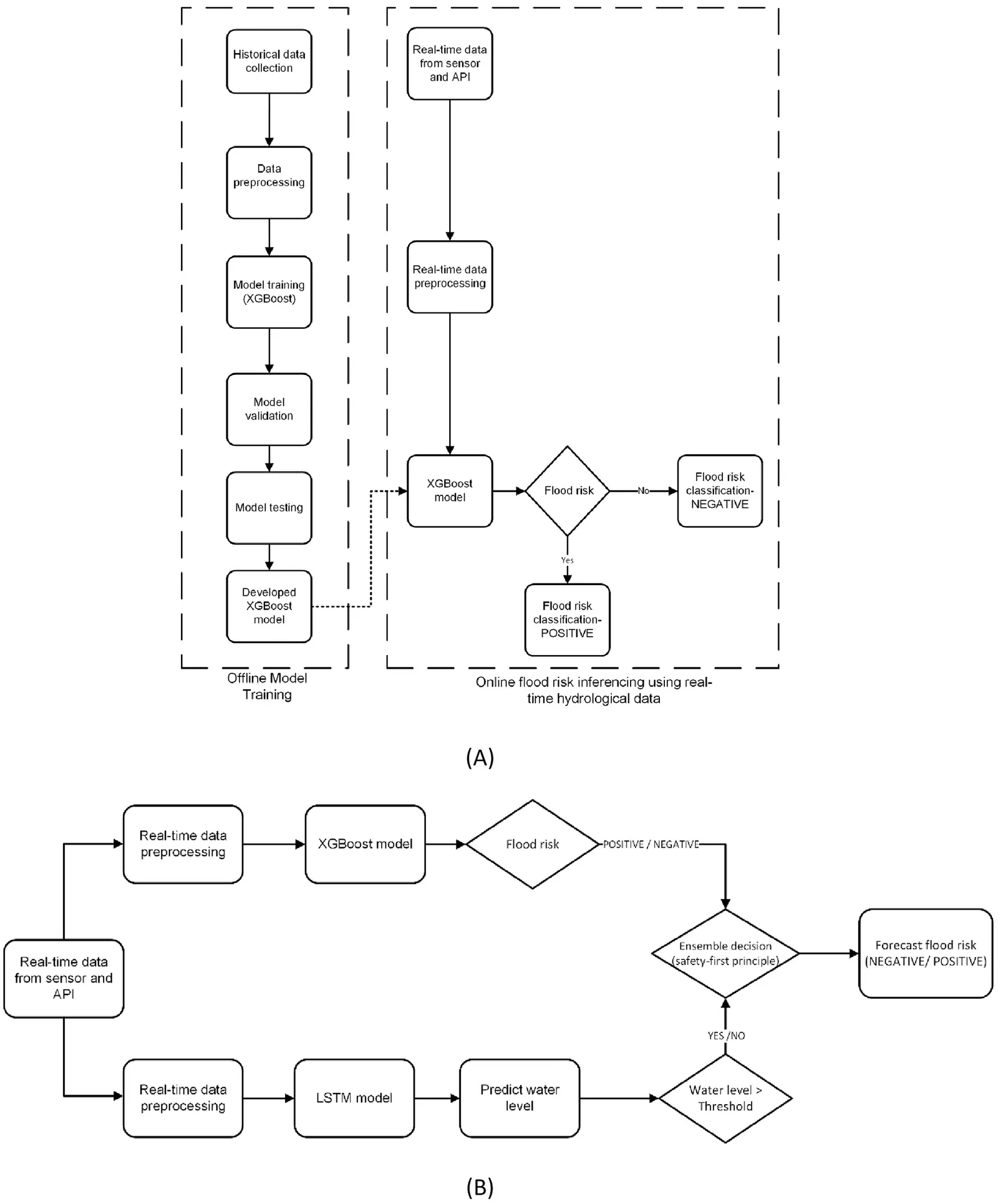
**(A)** Single-model (XGBoost) flood risk classification. **(B)** Two-model ensemble method (LSTM and XGBoost).

#### 4.2.4 Method 3: two-model combined rule-based ensemble

Unlike previously reported LSTM-XGBoost frameworks, which combine the models through sequential integration or statistical ensemble approaches, the proposed framework uses forecasting and classification models independently and combines their outputs through a rule-based decision-level fusion strategy. This hybrid approach combines the complementary characteristics of the two models for flood-risk assessment. This process is illustrated in Figure 4B.

- Components: It uses the LSTM network for water level forecasting and the XGBoost classifier for direct risk classification.
- Rule-Based Ensemble Logic: The outputs were combined using a rule-based decision-level fusion approach where the binary outputs of the LSTM and XGBoost models used a logical-OR decision rule, as shown in Equation 10. If both the LSTM-derived risk and the XGBoost classification agreed, that result was accepted. Otherwise (if the models disagreed), the system outputs the highest risk level - POSITIVE. This implements a safety-first rule in which a positive output from either model results in a positive ensemble decision, thereby prioritizing positive-class detection.

$$\begin{array}{l} P_{final}=\{\begin{array}{l} P_{LSTM}\;,\;P_{LSTM}=P_{XGB}\\ POSITIVE,\;P_{LSTM}\neq P_{XGB} \end{array} \end{array}$$ (10)

Where: *P*_*final*_is the final rule-based ensemble decision (“POSITIVE” or “NEGATIVE”), and *P*_*LSTM*_, *P*_*XGB*_ are the independent decisions from the LSTM and XGBoost models, respectively.

### 4.3 Model evaluation and validation

The dataset was imbalanced as there were significantly fewer flood events than non-flood events. This scenario was given specific attention to the model training. In order to make sure enough time is given before a predicted flood event; the models were tested by feeding data from varying number of lengths. For a comprehensive analysis, input windows of 1, 7, 14, and 21 days were tested to determine the ideal predictive window. Furthermore, a simple current-water-level threshold benchmark analysis was performed to assess whether the proposed rule-based ensemble method could provide predictive skill beyond the current river stage alone. The benchmark predicted the next-day flood risk as POSITIVE if the current Kaliodai River water level was equal to or greater than the threshold of 3.0 m, and as NEGATIVE otherwise. The benchmark was evaluated using the same chronological test observations for a one-day configuration of the proposed ensemble method. The accuracy, positive-class precision, positive-class recall, and positive-class F1-score were calculated using the same definitions applied in the proposed model.

#### 4.3.1 Critical metric

False negatives, which represent positive-labeled observations incorrectly classified as negative, are particularly important in flood forecasting as missed flood-risk conditions may delay warnings and increase potential consequences. A way to minimize false negatives is to maximize the positive-class recall (Equation 11). Hence, positive-class recall was selected as the key performance indicator (KPI) for the system. Recall was prioritized while maintaining the overall accuracy of the system. Therefore, within this study, a model with higher overall accuracy (Equation 12) but poor positive-class recall was considered less suitable for the safety-oriented flood-risk classification objective.


$$\begin{array}{l} Recall=\;\frac{TP}{TP+FN} \end{array}$$ (11)

$$\begin{array}{l} Accuracy=\;\frac{TP+TN}{TP+TN+FP+FN} \end{array}$$ (12)

Where: *TP* is true positives (correctly classified positive-labeled observation), *FN* is false negative (positive-labeled observation incorrectly classified as negative), *TN* is true negatives (negative-labeled observation correctly classified as negative), and *FP* is false positive (negative-labeled observation incorrectly classified as positive).

Considering the notable class imbalance in the available dataset, positive-class precision (Equation 13) and F1-score (Equation 14) were reported in addition to providing a comprehensive evaluation of the flood-risk classification performance. While the precision values indicate the proportions of positive predictions that correspond to actual positive observations, the F1-score is the harmonic mean of precision and recall.


$$\begin{array}{l} Precision=\;\frac{TP}{TP+FP} \end{array}$$ (13)

$$\begin{array}{l} F1=2\;\times \frac{Precision\;\times Recall}{Precision+Recall} \end{array}$$ (14)

The results report these metrics for the POSITIVE class, as the primary objective of the proposed safety-first system was to detect flood-risk conditions while minimizing missed positive events.

#### 4.3.2 Prediction window

This study analyzed multiple input window configurations to evaluate the sensitivity of model performance to the length of historical temporal information. Input windows of 1, 7, 14, and 21 days were tested using the chronological holdout. The window-size analysis was used as a comparative exploratory sensitivity analysis, with the input-window length being the modeling configuration varied across experiments. The LSTM architecture and training hyperparameters, XGBoost configuration, predictor variables, classification threshold, SMOTE procedure, and rule-based ensemble logic were kept fixed across the evaluated input window configurations. The chronological holdout was used to compare the observed performance of the different input window configurations and identify the configuration with the most favorable performance. The model fitting was performed using the chronological training portion. The holdout was subsequently used to test the already-developed modeling framework under different input-window configurations.

### 4.4 IoT hardware design and implementation

Figure 5 shows the hydrological data sensing layer, which comprises an IoT sensor node powered by a 12 V battery connected to a solar panel. The node comprises a rain gauge and an ultrasonic sensor. The sensor data are transmitted to the cloud via a wireless medium for AI decision-making, storage, and visualization.

**Figure 5:**
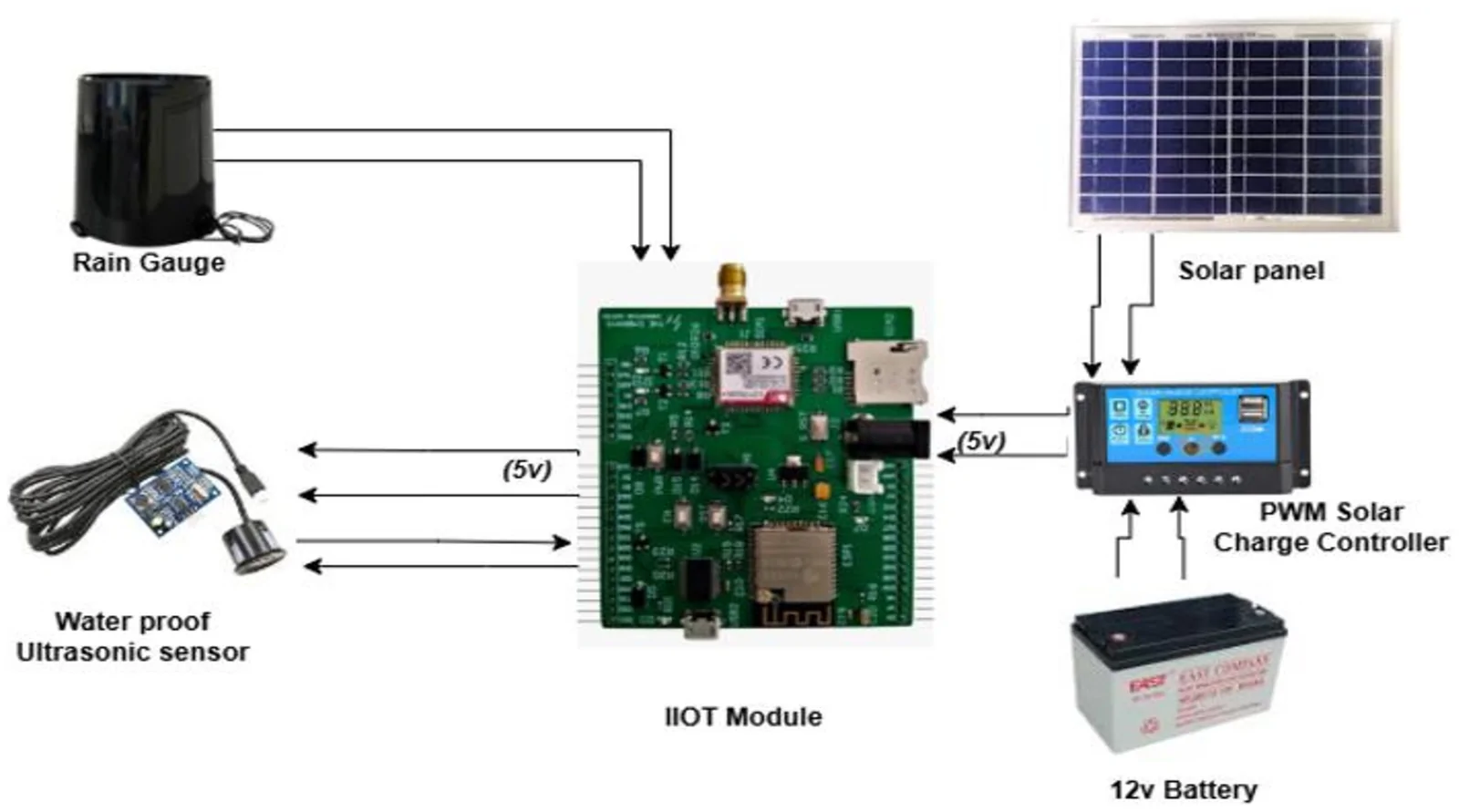
Block diagram of the installed hardware.

#### 4.4.1 Sensor integration and microcontroller

The IoT controller is an ESP32-based module with NB-IoT and Wi-Fi connectivity, which acts as both a processor and a gateway. The sensors were interfaced with the controller input ports.

- Water level sensor: A JSN SR-04T waterproof ultrasonic sensor was used to measure the river level. The water level was calculated by measuring the ultrasonic pulse travel time.
- Rain gauge: A XS-RS01 tipping-bucket rain gauge was connected to the ESP32 through a reed-switch input. Each bucket tip was counted by the ESP32, with each tip corresponding to 0.3 mm of rainfall. The accumulated tip count was converted to rainfall depth and reset at midnight, providing a calendar-day accumulated rainfall value for transmission to the IoT dashboard.
- Renewable energy: A 12 V solar panel connected to a pulse width modulation (PWM) solar charger controller regulates a 12 V rechargeable battery. The battery serves as the main power source for the system.
- Power distribution: The PWM solar controller provides a regulated 5 V output to power the microcontroller and the ultrasonic sensor.
- Data collection and transmission: The IoT microcontroller serves as a data aggregator and communication gateway. The system continuously collects and processes data from both sensors.

The processed data are transmitted through a wireless connection to a cloud platform, either via cellular NB-IoT or Wi-Fi internet, depending on the availability of connectivity. All components were integrated into a weatherproof housing to ensure signal integrity and robust operation in outdoor environments. Figure 6 shows the IoT hardware configuration of the deployed system.

**Figure 6:**
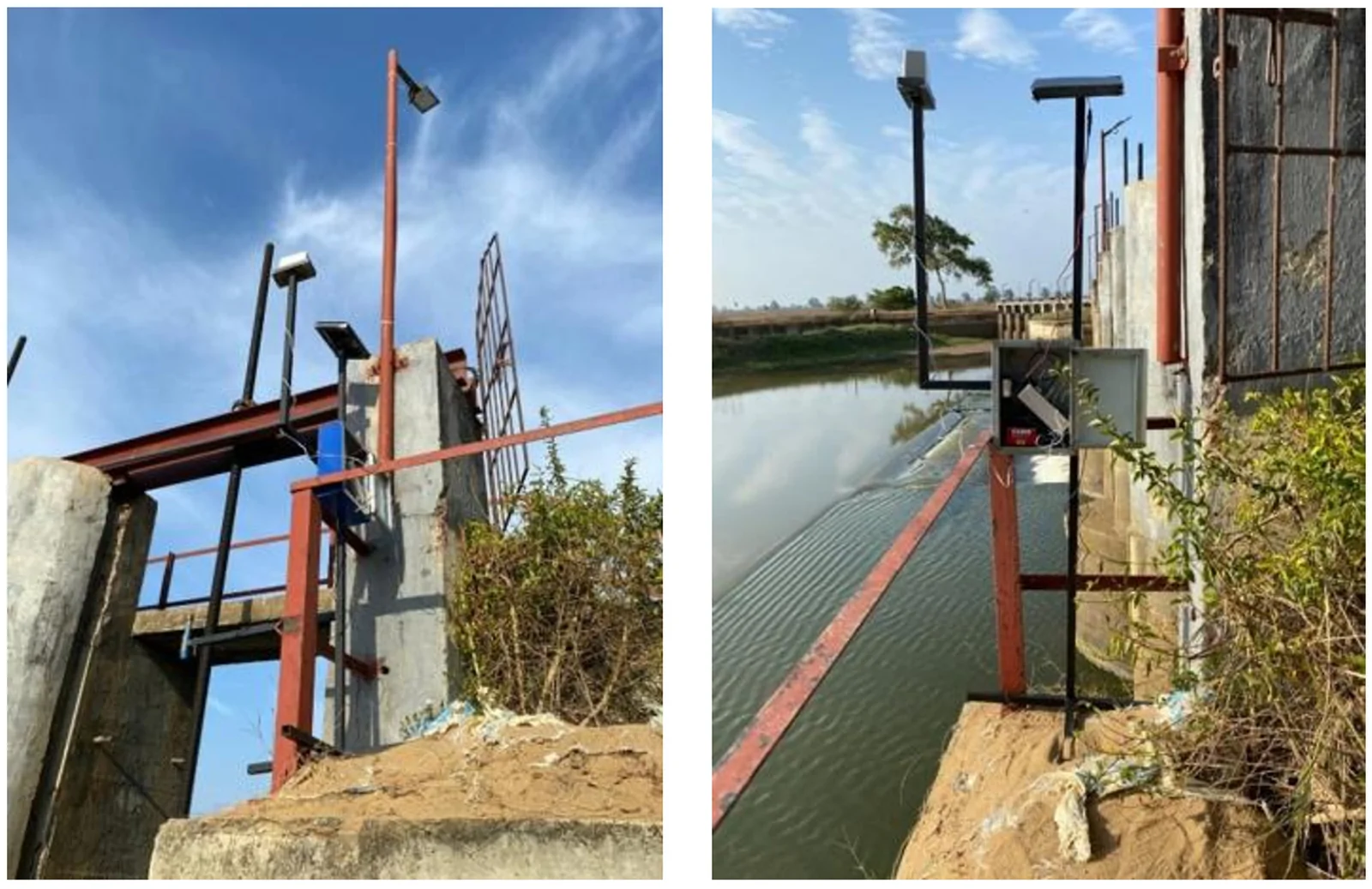
Installed hardware along the downstream of the Gal Oya River.

### 4.5 Data acquisition, management, and visualization

In AI-based flood forecasting systems, cloud connectivity and data visualization play crucial roles in providing real-time monitoring and decision support.

#### 4.5.1 Data acquisition and integration

The data stream includes real-time sensor data and supplementary hydrological data from upstream for comprehensive analysis of the river basin's dynamics. The deployed IoT sensor node transmits downstream rainfall and river water level data in real-time to the cloud via secure message queuing telemetry transport (MQTT) protocols, while upstream catchment rainfall, dam water level, and dam discharge rate are obtained from public Application Programming Interfaces (API) provided by the Department of Irrigation and the Department of Meteorology of Sri Lanka. During the operational inference, the prediction was made using the latest available downstream observations along with available upstream hydrological information. The catchment rainfall was retrieved as the preceding day's completed daily precipitation total. The deployed LSTM inference used a 1-day input configuration, consisting of one daily predictor timestep and five predictor variables.

#### 4.5.2 Cloud platform and data management

The integration of cloud storage and cloud-based prediction layer provides scalability and real-time access for the proposed system. For proof of concept, the ThingsBoard Community Edition IoT cloud platform was integrated into the system as the IoT cloud. In addition, the IoT cloud provides native device management, access control, and analytics, which are essential for scaling of the system.

#### 4.5.3 Data visualization

The system uses a web-based dashboard to display the latest hydrological data from the sources and decision layer forecast. The following key parameters are displayed, as shown in Figure 7.

- Date of measurement
- Forecasted flood risk for the next day as “Flood Warning”: NEGATIVE or POSITIVE
- Predicted water level for the next day
- Observed rainfall values from both downstream sensors and upstream catchments
- Current river water level
- Dam water height and discharge rate

**Figure 7:**
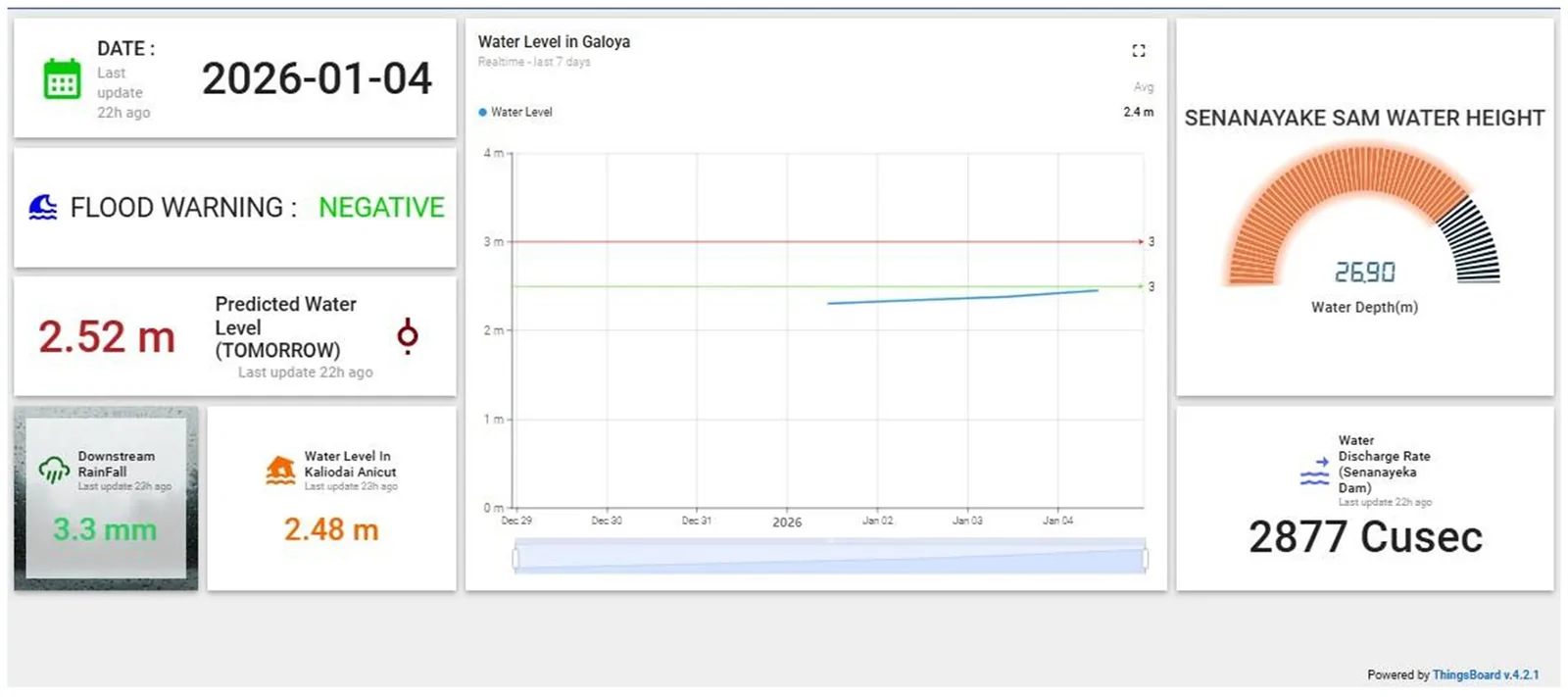
Dashboard for data visualization.

### 4.6 An integrated model of AI-based flood risk classification and water level forecasting system

Figure 8 shows an integrated overview of the complete system from model development to forecasting, grouped into three layers: model training, hardware, and software.

**Figure 8:**
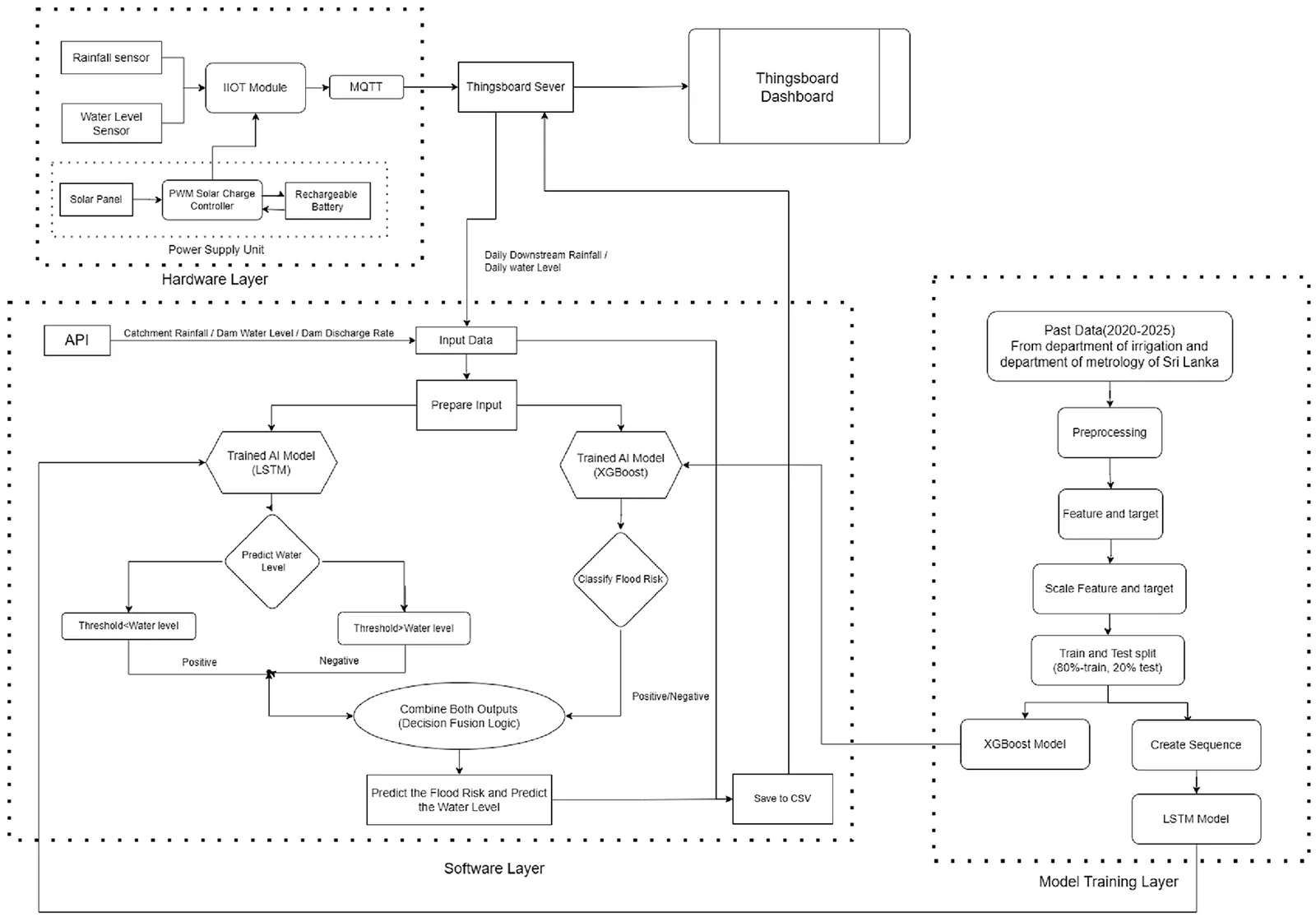
An integrated model of AI-based flood risk classification and water level forecasting tested in this study.

The model training layer highlights the key steps involved in the model development stage. Historical hydrological data from 2020 to 2025 were used to train and validate the models. The LSTM model was trained to predict water levels, while the XGBoost model was trained to predict flood risk levels, together with the flood-risk indication.

The hardware layer handles data acquisition, where real-time sensor data fundamentally initiate the forecasting process.

The software layer acts as the brain of the system, where the sensor data and historical hydrological data are processed to generate flood predictions. The layer also handles the preprocessing, normalization, feature extraction, and scaling of the inference data before feeding them to the two trained models from the model training layer. The LSTM model forecasts the next-day river water level, while the XGBoost classifier predicts the flood risk category. The outputs of these models are combined through a decision-fusion logic (“safety-first” principle) to support flood-risk decision making while prioritizing positive-class detection.

The inferencing outputs of the models are presented as visual data via a browser-based dashboard, supporting informed decision-making. The integration of AI and IoT through three layers provides a framework for real-time river water level monitoring and flood risk prediction.

## 5 Results

This section presents the predictive performance results obtained from the labeled historical dataset and operational observations during real-time field deployment. The quantitative evaluation of the machine-learning models was performed using the historical dataset, whereas the field deployment was used to demonstrate operational feasibility under the observed field conditions. An analysis of the performance of the models against varying temporal data window sizes was performed individually for each model, as well as for the rule-based ensemble model.

The model performance was evaluated using overall accuracy, negative-class recall, positive-class precision, positive-class recall, and positive-class F1-score. Although positive-class recall was considered the primary performance indicator, considering the criticality of a missed flood event for an early warning system, precision and F1-score were reported to provide a more balanced assessment, given the imbalanced nature of the class distribution. These details are presented separately in tables for easy comparison. The subsequent section presents the sample data collected from the real-world demonstration for up to 10 days. Data from days where significant rainfall and catchment water release are presented for understanding various hydrological and environmental conditions of the study location.

### 5.1 Exploratory window-size sensitivity analysis

Table 4 presents a comparison of the standard metrics of each individual model and the rule-based ensemble model for varying lengths of temporal data. This analysis aimed to examine the sensitivity of the model performance to the length of historical input data. The same chronological holdout datasets were maintained for evaluating all configurations. Hence, the comparison is presented as an exploratory window-size sensitivity analysis. With a 21-day window size, the LSTM model achieved a maximum overall accuracy of 98.72%, while the XGBoost model achieved an accuracy of 98.08%. Although there was a very small difference in overall accuracy between these models, the XGBoost model provided better recall performance, indicating higher sensitivity to flood-labeled events in the study. It was observed that the rule-based ensemble model showed performance improvements in recall and overall stability across varying window sizes, even when the window size was reduced.

**Table 4:** Model performance across different window sizes.

Model	Window size	Overall accuracy	Recall (Neg.)	Recall (Pos.)
LSTM	7 days	0.9714	0.9807	0.2500
	14 days	0.9744	0.9838	0.2500
	21 days	0.9872	1.0000	0.0000
XGBoost	7 days	0.9810	0.9839	0.7500
	14 days	0.9808	0.9903	0.2500
	21 days	0.9776	0.9838	0.5000
Ensemble (LSTM + XGBoost)	7 days	0.9778	0.9775	1.0000
	14 days	0.9776	0.9806	0.7500
	21 days	0.9712	0.9740	0.7500

#### 5.1.1 Exploratory evaluation of small window sizes

Table 5 summarizes the results of the tests performed using the rule-based ensemble method with progressively shorter windows, from 7 days to 1 day. For the one-day configuration, the test set included 316 samples, out of which 312 were negative-labeled samples (no flood) and 4 positive-labeled samples. The four positive-labeled samples in the test set occurred between 25 and 28 November 2024, representing one continuous positive-labeled flood episode. These samples were labeled positive as their corresponding observed water levels met or exceeded the 3.0 m empirical local threshold defined in the study.

**Table 5:** Performance of the rule-based ensemble model (LSTM + XGBoost) across shorter input window sizes.

Window size	Test samples	Overall accuracy	Recall (Neg.)	Precision (Pos.)	Recall (Pos.)	F1-score (Pos.)
7 Days	315	0.9778	0.9775	0.3636	1.0000	0.5333
6 Days	315	0.9746	0.9743	0.3333	1.0000	0.5000
5 Days	315	0.9746	0.9743	0.3333	1.0000	0.5000
4 Days	315	0.9778	0.9775	0.3636	1.0000	0.5333
3 Days	316	0.9747	0.9744	0.3333	1.0000	0.5000
2 Days	316	0.9778	0.9776	0.3636	1.0000	0.5333
1 Day	316	0.9873	0.9872	0.5000	1.0000	0.6667

As shown in Figure 9, the developed rule-based ensemble model classified four positive-labeled samples (TP = 4, FN = 0), while producing four false-positive predictions (FP = 4). The 1-day configuration achieved an overall accuracy of 98.73%, positive-class precision of 50.00%, positive-class recall of 100%, and positive-class F1-score of 66.67% on this chronological holdout. These values represent the observed performance for the evaluated holdout.

**Figure 9:**
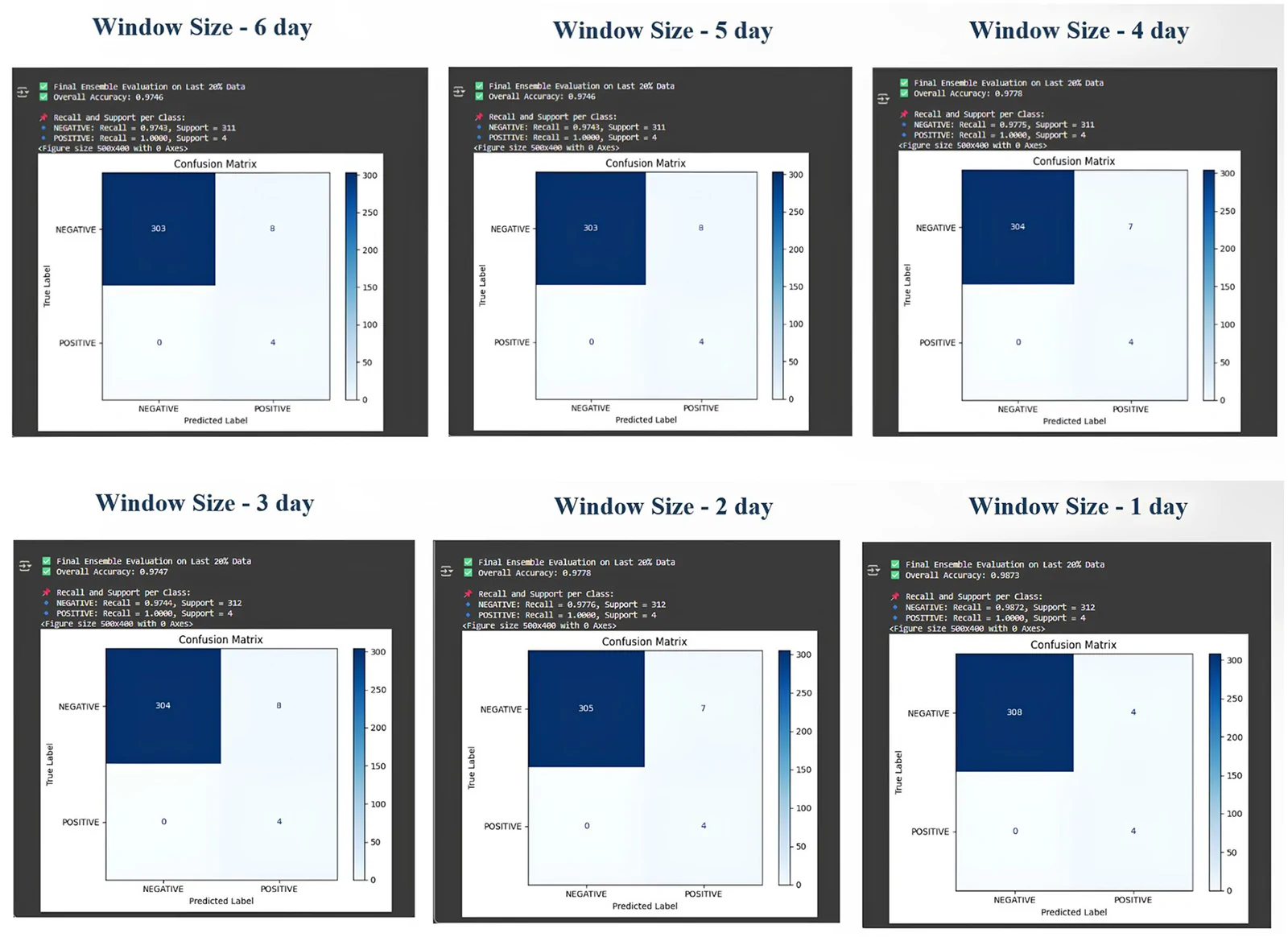
Performance of rule-based decision level-ensemble logic under varying days-window size configurations.

Given that only four positive-labeled observations were present in the test set, the uncertainty associated with the positive-class performance estimates was evaluated by calculating confidence intervals. The exact binomial (Clopper-Pearson) method was used at the 95% confidence level for positive-class recall and positive-class precision. The positive-class recall was 100.0% (95% CI: 39.8%−100%), based on four correctly classified positive observations out of four. The positive-class precision was 50.0% (95% CI: 15.7%−84.3%), based on four true-positive predictions among eight positive predictions. The wide confidence intervals highlight the substantial statistical uncertainty associated with the reported performance estimates. Hence, the reported 100% positive-class recall should be interpreted as a sample-level classification result based on four positive-labeled observations belonging to one continuous flood episode, rather than evidence that the population-level recall for flood detection is 100%.

The rule-based ensemble was able to maintain 100% positive-class recall across the seven evaluated window sizes. However, the precision varied from 33.33% to 50.00%, indicating the varying number of false-positive predictions. Among the tested configurations, the one-day input window configuration showed the most favorable observed balance of performance metrics among the configurations achieving 100% positive-class recall. It achieved an overall accuracy of 98.73%, positive-class precision of 50.00%, and F1-score of 66.67% on the chronological holdout.

#### 5.1.2 Comparison of rule-based ensemble with a current-water-level threshold benchmark

Table 6 presents the performance results of the rule-based ensemble method compared with a simple current-water-level threshold benchmark. The evaluation was performed in order to determine if the proposed rule-based ensemble method provides predictive skill beyond the current river stage alone. The current-water-level benchmark achieved 99.37% overall accuracy, 75.00% positive-class precision, 75.00% positive-class recall, and 75.00% positive-class F1-score. The proposed rule-based ensemble method with a 1-day input window achieved 98.73% accuracy, 50.00% positive-class precision, 100.00% positive-class recall, and 66.67% positive-class F1-score. Both methods were evaluated using the same chronological test set of 316 observations. The benchmark correctly classified three out of the four positive-labeled observations while missing one positive observation on November 25, 2024, where the previous day water level was 2.9 m (2,900 mm). The proposed rule-based ensemble correctly classified all four positive-labeled observations but produced four false-positives. Hence, the proposed ensemble provided a higher observed positive-class recall, whereas the simple current-water-level benchmark provided higher precision, F1-score, and overall accuracy for the same test set.

**Table 6:** Comparison of rule-based ensemble with current-water-level threshold benchmark.

Method	Accuracy	Positive precision	Positive recall	Positive F1
Current-water-level threshold	99.37%	75.00%	75.00%	75.00%
Rule-based ensemble with 1-day input window	98.73%	50.00%	100.00%	66.67%

### 5.2 Gain-based feature importance analysis for the XGBoost classifiers

Table 7 presents the gain-based feature importance analysis extracted from the trained XGBoost model. The results indicated that for the selected study location, river water level was the most influential predictor, with a gain metric of 156.926285. This observation is consistent with the literature. The river water level was followed by the reservoir water level, with a gain value of 17.619219. Downstream and catchment rainfall had gain values of 1.852051 and 0.885171, respectively, whereas reservoir discharge rate showed negligible importance.

**Table 7:** Gain-based feature importance analysis for the XGBoost classifier.

Feature	Gain
Water level (Kaliodai)	156.926285
Reservoir water level	17.619219
Downstream rainfall	1.852051
Catchment rainfall	0.885171

### 5.3 LSTM regression performance

The LSTM model performance metrics are listed in Table 8. The LSTM model achieved an RMSE of 327.07 mm and MAE of 233.11 mm on the evaluated data. The *R*^2^ and NSE values were both 0.8023, indicating that the model captured a substantial proportion of the observed variation in river water level within the evaluated dataset.

**Table 8:** LSTM model evaluation metrics.

Metric	Value
RMSE	327.07 mm
MAE	233.11 mm
*R^2^*	0.8023
NSE	0.8023

### 5.4 Real-time field deployment and operational demonstration

Table 9 presents a set of samples of real-world data and the corresponding flood risk classifications. The data were collected by deploying the developed IoT node at a suitable location in the downstream region of the Gal Oya River and from public data sources.

**Table 9:** Field-collected real-time hydrological data and predicted flood-risk classification.

Date	Downstream rainfall (mm)	Water level (m)	Water depth (sena dam) (m)	Spilling (sena dam) cusec	Catchment rainfall (mm)	Predicted water level (LSTM) (m)	Flood risk
21/11/2025	9	0.3	23.165	0	16.5	0.423	0
23/11/2025	0	0.38	23.195	0	22.4	0.477	0
24/11/2025	23.4	0.62	23.195	0	50.5	0.847	0
25/11/2025	80.4	2.1	23.287	0	24.4	2.106	0
26/11/2025	156.3	2.25	23.744	0	47.6	2.409	0
29/11/2025	2.1	2.6	29.261	0	93.6	2.32	0
01/12/2025	0	2.4	29.535	4,480	0.4	2.636	0
01/01/2026	0	2.34	26.883	2,877	15.4	2.409	0
02/01/2026	2.1	2.38	26.853	2,872	22	2.44	0
03/01/2026	3.3	2.48	26.883	2,877	3.5	2.523	0

Moderate, heavy, and extreme downstream rainfall was recorded on November 24, 25, and 26, 2025, respectively. On these 3 days, the model was able to predict downstream river water-level changes that corresponded with the observed increase in water level. On November 29, 2025, the observed downstream water level was 2.60 m, while the LSTM predicted 2.32 m, corresponding to an absolute error of 0.28 m and a percentage error of 10.77%, calculated as in Equation 15:


$$\begin{array}{l} Percentage\;Error=\frac{\left|H_{obs}-H_{pred}\right|}{H_{obs}}\times 100 \end{array}$$ (15)

Where: *H*_*obs*_ and *H*_*pred*_ represent the observed and predicted water levels, respectively.

The last three rows of the tables show that even during periods with minimal or zero downstream rainfall, the spilling of the main dam contributed to the water level increase at the downstream, which is reflected by the water level prediction. The field observations demonstrate that the LSTM inference pipeline produced water-level estimates that followed the observed variations during the selected deployment periods. Another significant prediction of the proposed framework is flood risk classification, which is given in the last column of the table. The flood-risk output was NEGATIVE, indicated by zero value, which was consistent with observed real-world conditions during deployment days. Also, the predicted water levels were consistent with the LSTM regression metrics shown in Table 8.

## 6 Discussion

### 6.1 Performances of LSTM and XGBoost ensemble models in flood forecasting

This study proposes a river flood forecasting framework based on the integration of AI and IoT. The rule-based ensemble model uses LSTM and XGBoost models to improve inference capabilities. Real-time hydrological data were collected using IoT sensors and through the public API of governmental hydrological data sources. Although many ML models have been used for flood forecasting studies, this system integrates a rule-based decision-level fusion model, IoT, and public hydrological data sources to provide an integrated approach to flood forecasting. Although the LSTM model achieved a good overall accuracy (98.72%) with a 21-day input window, the corresponding recall positive (flood) case was 0.0, indicating that all positive-labeled samples were misclassified as non-flood samples, highlighting that accuracy was not always the best metric for evaluating highly imbalanced flood datasets available for this study. In safety-critical applications such as flood forecasting, false negatives can have greater consequences than false alarms. Hence, recall was selected as the primary metric, and the rule-based ensemble model was designed to prioritize positive-class detection while maintaining overall accuracy.

The XGBoost method showed relatively low temporal stability as evidenced by the significant fluctuation in its recall for flood events (Recall Pos.), which varied between 0.25 and 0.75 across the 7, 14, and 21-day window sizes (Table 4). Comparative tests showed that the rule-based ensemble model provided higher observed positive-class recall than individual models and simple current-water-level threshold benchmark on the same chronological holdout. Based on the observations specific to the present dataset and case study, the results are consistent with the expected roles of the two models within the evaluated dataset; the LSTM model provided a mechanism for learning temporal patterns and the XGBoost provided effective classification performance for the evaluated flood-risk task. Further longitudinal studies using larger and more diverse datasets may be required for the broader applicability of these tested models.

### 6.2 Benchmark comparison of the proposed framework

Among the evaluated short-window configurations, the rule-based ensemble model with a 1-day input window showed the most favorable observed performance, achieving an overall accuracy of 98.73%, positive-class precision of 50.00%, positive-class recall of 100%, and a positive-class F1-score of 66.67% on the chronological holdout. Although the model predicted four negative observations as positive, it correctly classified all four positive-labeled observations in the test set corresponding to one continuous positive-labeled flood episode occurring from 25 to 28 November 2024. This reflects the intended safety-first logical-OR decision-level fusion strategy, which prioritizes minimizing missed positive flood conditions at the expense of additional false alarms. Hence, interpreting the observed 100% positive-class recall within the given case study and dataset, four positive-labeled test samples were correctly detected. However, further studies are required to establish this as evidence of a generalized flood detection capability across four independent flood events. Furthermore, the higher F1-score for the 1-day configuration indicates the balance between flood detections and false-positive detections among the evaluated other window configurations.

Within the evaluated dataset, for the rule-based ensemble, configurations with shorter input window sizes showed a favorable observed classification performance compared with longer windows. This observation suggests that recent hydrological observations contain useful predictive information for the next-day flood-risk classification for the study location. However, it does not establish that short-term hydrological variations are hydrologically dominant over long-term temporal effects. An observation from the XGBoost feature importance analysis showed that reservoir water discharge had very negligible importance. The predictive information of the reservoir discharge may already be represented by the correlated variable, observed river water level, in the specific dataset and model. Hence, the interpretations are specific to the specific dataset and hydrological conditions, and the significance of reservoir discharge in flood prediction needs to be further established for the study location.

The benchmark comparison provides an important interpretation of the proposed framework. The current water level at the Kaliodai River was the dominant XGBoost predictor, and the simple current-water-level threshold achieved an accuracy of 99.37%, precision of 75.00%, positive-class recall of 75.00%, and F1-score of 75.00% for the same chronological test observations. While the simple current-stage threshold may have superior classification performance over the proposed framework, the rule-based ensemble achieved higher observed positive-class recall, correctly identifying the positive observation on 25 November, 2024 when preceding day's water level was 2.9 m. From the evaluated test cases, it is observed that the ensemble's additional model-based information and safety-first decision rule was associated with higher observed sensitivity to the onset of the positive-labeled episodes. Yet, the tradeoff was the four false positives compared with one for the simple threshold benchmark.

### 6.3 Operational feasibility of the proposed ensemble model framework for flood forecasting

The developed rule-based ensemble model was integrated with the IoT-based monitoring system, which collected real-time field data from the Gal Oya River basin of Sri Lanka, to demonstrate the practical operation of the proposed framework. The predicted water levels generally followed the observed river water level trends during the selected deployment periods, which included both relatively high and low rainfall conditions. The system also operated under conditions in which downstream rainfall was minimal, demonstrating that the monitoring and inference pipeline could process varying, real-time hydrological inputs. These observations demonstrate the operational feasibility of the proposed framework, including field sensing, public hydrological data integration, real-time data transmission and model inference.

Field deployment demonstrated the operational feasibility of implementing the proposed AI-IoT monitoring and inference framework. Although many existing studies have demonstrated the application of AI for flood forecasting, some have focused primarily on historical data, while relatively few have addressed real-time data integration with rule-based ensemble approaches. In addition, the emphasis on recall as the primary performance metric for the rule-based ensemble model was intended to reduce missed positive-labeled observations within the evaluated dataset.

### 6.4 Limitations of the study and future research directions

The key limitations of this study are highlighted below, which will help in understanding the factors when adapting the study method to other contexts and generalizing the findings.

(1) Limitations in the class imbalance mitigation strategy: SMOTE was only applied to the XGBoost classifier, whereas LSTM was trained using the original sequential data without oversampling, as conventional SMOTE was likely to disrupt the temporal dependencies inherent in time sequence data. Hence, the comparative performance of individual models may have been influenced by the different imbalance handling strategies in addition to the inherent model architectures. Future work will investigate sequence-aware imbalance handling techniques for recurrent neural networks to enable a more consistent comparison.
(2) Limitations on the chronological holdout: The model performance was evaluated using a single chronological 80/20 train-test split. The LSTM and XGBoost models were fitted using the chronological training portion, while the chronological holdout was subsequently used to compare the varying input-window configurations. The 1-day configuration was identified as the most favorable observed configuration based on this comparative testing. Since the same chronological holdout was used to compare the different input window configurations, the results represent an exploratory observed sample-level performance rather than an independent estimate of generalization performance. Although this approach was chosen to preserve the temporal structure of the time-series data and avoid information leakage, the limited number of positive-labeled events in the test set introduces substantial uncertainty into the estimated positive-class performance metrics, particularly recall. Hence, the reported performance results should be interpreted as observed performance on the available test observations rather than population-level estimates. Future work must address this limitation by separating temporal model-selection data from an untouched final test period and using repeated or rolling-origin time-series validation to select input window configurations and assess generalization. Furthermore, the 80/20 train-test split with 10% validation split was important in the LSTM model to prevent overfitting. However, advanced regularization techniques such as dropout and early stopping were not employed in the current study. Instead, model complexity was managed through a relatively simple LSTM architecture and limited training epochs. Future work will investigate these regularization methods using larger datasets.
(3) Limited number of independent flood episodes in test set: In this study, one continuous positive-labeled flood episode occurred from November 25 to 28, 2024. Although all four positive-labeled observations were correctly classified, the metrics reported are based on only four samples from a single positive episode. The reported 100% positive-class recall does not imply that four independent flood episodes were detected, or that the model would achieve 100% recall across a broader population of flood observations or independent flood episodes. Future studies should evaluate the model using larger datasets containing a greater number of independent flood episodes to assess event-level generalization more robustly.
(4) Limited real-time observation period: Real-world testing has been a challenge with a limited observation period and absence of flood events during field deployment. Even though the system correctly classified negative for all 10 observed real-world observations, these observations need to be further validated with the positive-class recall or flood-event detection under actual flood conditions, as no positive flood event occurred during this observation period. Therefore, the field deployment demonstrated operational feasibility under observed conditions rather than predictive validation under real-world flood conditions.
(5) Using prototype hardware: The study was limited to small-scale IoT deployment using prototype sensors. Future work will focus on sensor calibration, scaling of the IoT nodes, sensor failure detection mechanisms and edge device-based inference capabilities.

## 7 Conclusions

Most existing conventional flood monitoring and forecasting systems rely on manual measurements and threshold-based sensors. Further, logistical and technological constraints in real-time monitoring pose significant challenges in the accurate forecasting of flood events. This study presents the design and development of an AI-IoT-based system for river water-level monitoring and flood forecasting, with its practical operation demonstrated through field deployment. The proposed system integrates IoT with ML models for real-time flood-risk forecasting. Hydrological data obtained from field-deployed sensors and public data sources are processed using a rule-based ensemble model that combines LSTM and XGBoost models.

A comparative evaluation of the input-window configurations on the chronological holdout showed that the rule-based ensemble model with 1-day input window provided the most favorable observed balance of performance metrics with 98.73% overall accuracy, 50.00% positive-class precision, 100% positive-class recall, and 66.67% positive-class F1-score. These results should be interpreted as the observed performance from the exploratory window-size analysis rather than an independent estimate of generalization performance, considering that the same chronological holdout was used for the window-size comparison and identification of the one-day configuration. All four positive-labeled observations in the holdout test set were correctly classified, representing one continuous positive-labeled flood episode and sample-level recall performance for the available test observations.

Furthermore, the operational feasibility of the system was demonstrated with real-time field deployment in the river flood-prone downstream regions of the Gal Oya River in the eastern part of Sri Lanka. The field deployment demonstrated real-time sensing, data transmission, preprocessing, model inference, and dashboard visualization. However, as no flood events occurred during the deployment period, the field validation of flood-event detection or positive-class recall under actual flood conditions needs to be established in future studies. Therefore, the quantitative predictive performance reported in this study was evaluated using a historical labeled dataset, whereas field deployment demonstrated operational feasibility.

The main contributions of this study include the following: (a) The development of a fully integrated AI–IoT-based flood forecasting system for real-time water level monitoring and prediction; (b) the implementation of a rule-based ensemble learning approach that combines the temporal modeling capability of LSTM with the classification robustness of XGBoost; and (c) the demonstration of operational feasibility of the proposed system using real-time field data.

Future research will incorporate the use of transformer-based architectures for forecasting, increased robustness using Shapley additive exPlanations (SHAP) analysis, and explainable AI techniques for enhanced interpretability. Further validation of the system could be conducted through longer-term deployment, expansion of the sensor network to multiple locations, and testing across different river environments. In addition, the integration of complementary geospatial and hydrological datasets, including digital elevation models (DEM), soil characteristics, rainfall stream network information, and remote sensing data, can be investigated to further enhance the performance and robustness of the proposed framework.

## Data Availability

The original contributions presented in the study are included in the article/Supplementary material, further inquiries can be directed to the corresponding author.

## Footnotes

1
https://www.dmc.gov.lk/index.php?option=com_dmcreports&view=reports&Itemid=277&limit=0&search=&report_type_id=6&fromdate=&todate=&lang=en

## Supplementary material

Graphical abstract An integrated model tested in the study.
